# Chloroanisoles and Chlorophenols Explain Mold Odor but Their Impact on the Swedish Population Is Attributed to Dampness and Mold

**DOI:** 10.3390/ijerph17030930

**Published:** 2020-02-03

**Authors:** Johnny C. Lorentzen, Stephanie A. Juran, Lena Ernstgård, Mats J. Olsson, Gunnar Johanson

**Affiliations:** 1 Institute of Environmental Medicine, Integrative Toxicology, Karolinska Institutet, 171 77 Stockholm, Sweden; sjuran@web.de (S.A.J.); lena.ernstgard@ki.se (L.E.); gunnar.johanson@ki.se (G.J.); 2 Department of Clinical Neuroscience, Karolinska Institutet, 171 77 Stockholm, Sweden; mats.j.olsson@ki.se

**Keywords:** mold, odor, sick building syndrome, building related illness, asthma, allergy, pesticides, biocides, wood preservatives, indoor air

## Abstract

We recently reported that mold odor may be explained by chloroanisoles (CAs) formed by microbial biotransformation of chlorophenols (CPs) in legacy wood preservatives. Here we examine psychophysical aspects of CAs and trace their historic origins in buildings. Our exposure of healthy volunteers shows that 2,4,6-triCA is often perceived as unpleasant, characterized as musty or moldy and is detected at 13 ng/m^3^ or lower. Similar concentrations are reported in buildings with odor complaints. Scrutiny of written records reveal that new building construction methods were introduced in the 1950s, namely crawlspaces and concrete slabs on the ground. These constructions were prone to dampness and attack from wood decay fungi, prompting chemical companies and authorities to advocate preservatives against rot. Simultaneously, CPs became household chemicals used for example in indoor paints. When large-scale odor problems evolved, the authorities that once approved the preservatives attributed the odor to hidden mold, with no evidence that substantial microbial biomass was necessary for odor formation. Thereby the public remained unaware of problematic exposure to CPs and CAs. We conclude that the introduction of inappropriate designs of house foundations and CP-based preservatives once ignited and still provide impetus for indoor air research on “dampness and mold”.

## 1. Introduction

Two studies have provided evidence that chloroanisoles (CAs) cause indoor air quality problems with musty [1] or moldy [2] odor. This raises the basic question why these volatile chemicals have been overlooked in past as well as present research on “dampness and mold” [3]. A place for CAs in this context seems logical as they are produced by microbes given access to dampness and chlorophenols (CPs) in legacy wood preservatives. CP-based preservatives were once used world-wide in buildings and elsewhere until they were banned in the 1970s and 1980s due to detrimental effects on environment and health. High exposure to CPs may lead to cancer [4] and cause acute intoxication in humans, cases have been described where people in homes have been poisoned [5]. CPs may also disrupt endocrine systems [6,7,8], affect the immune system [9,10] and may change the microbial flora of skin, gut, and airways as they do in soil [11,12,13]. Association of asthma with CP in drinking water has been reported [14]. That microbial biotransformation of CPs leads to odorous CAs raises the possibility of a long-lasting confusion of CPs and mold concerning health effects. Obviously, such confusion may have led to misguided and misinterpreted studies, regarding, for example, epidemiological associations between mold odor and inflammatory airway diseases. Confusion of CPs and mold could also be the explanation for the difficulties to identify relevant hazardous mold related agents, effect thresholds, and health-based exposure limits or guidance values [15].

In Sweden, building investigators and other field practitioners have used analyses of CPs/CAs since 1999. By compiling such analyses, we demonstrated the presence of CPs/CAs in both indoor air and construction materials from hundreds of problem buildings erected or renovated between 1955 and 1978, when CPs were permitted and promoted during an unprecedented building boom called the one million homes program [2]. Furthermore, CAs in many Swedish indoor environments are very odor potent as they often originate from a popular Swedish product called KP-Cuprinol once used to impregnate construction timber in, for example, homes, kindergartens, schools, and offices. KP-Cuprinol contains CPs, partly pentaCP (PCP) but mainly 2,3,4,6-tetraCP and 2,4,6-triCP. In damp conditions, these CPs are methylated by microbes into the corresponding 2,3,4,6-tetraCA and 2,4,6-triCA which are notorious in other contexts for deteriorating the smell of foods and beverages at very low concentrations. In fact, the odor thresholds are so low that a new mechanism of olfaction has been suggested for 2,4,6-triCA (3CA) [16].

An important question concerns if and how people perceive current low levels of 3CA. Only one study (conference paper) reports odor thresholds in air suggesting 5 ng/m^3^ for 3CA, as presented from an outlet port linked to a gas-chromatography mass-spectrometer [17]. We reported a mean indoor air concentration of 9 ng/m^3^ in quantified air samples from problem buildings, and performed a toxicological evaluation suggesting that these concentrations are not detrimental to human health per se, but sufficiently high to cause odor [2].

Altogether, it seems likely that CPs and CAs have caused significant indoor environment problems for many decades [2]. Further knowledge is beneficial for citizens and those working to ensure their good and safe environments, e.g., researchers, officials at health agencies, and practitioners.

In this study, we aimed to determine if the reported low indoor levels of CAs are detectable by olfaction, how they are perceived, and if they can be described as moldy. Furthermore, we aimed to identify and piece together information on why Swedish institutions once promoted preservatives in buildings but then disregarded the hazardous chemicals when indoor problems evolved.

## 2. Materials and Methods

### 2.1. Odor Exposure System

We developed an exposure method where 3CA is added to aluminum airbags thereby minimizing surface adherence of the notoriously “sticky” molecules and enabling stable concentrations in the low ng/m^3^ range to be presented through air outlets, as previously described [18]. In brief, nine lockable airtight bags with a maximum volume of 100 L were built of aluminum covered plastic foil (Skultuna Flexible AB^®^, Skultuna, Sweden) with nose adapters forming the air-outlet of the bags (silicone tube, glass, stainless steel, in-house made Teflon nostril piece) for active sniffing. The bags were filled with clean, carbon-filtered and odor-free compressor air. A micro-volume syringe was used to inject 3CA (CAS-Nr 87-40-1, LGCstandards, Borås, Sweden) dissolved in ethanol (LiChrosolv^®^, Merck, Darmstadt, Germany) and ultrapure water (Biochrom GmbH, Berlin, Germany). Sniffing from odor-bags was done in a standardized manner and blinded during odor detection experiments. After one ordinary inhalation–exhalation cycle, the two nose adapters were positioned tightly in each nostril for inhalation that lasted about three seconds.

### 2.2. Healthy Volunteers

Participants were recruited by advertising on public billboards at the Karolinska Institutet campus and homepage as well as on a local homepage for recruitment of test persons (www.studentkaninen.se). They were considered healthy and with normal olfactory function after an initial check comprising the following standardized physiological and psychological assessments: (a) Examination of blood pressure, heart- and lung function; (b) medical questionnaires used to exclude chronic diseases, any history of asthmatic symptoms, use of nicotine (smoking or chewing tobacco) antihistamines or other medication; (c) olfactory functioning test (Sniffin’ Sticks identification test, Burghart, Wedel, Germany, five item version) and the Symptom check list-90 [19]. Forty-four participants between 18 and 49 years of age, 31 females (mean 29 years) and 13 males (mean 28 years) participated, whereof 10 females (mean 32 years) and 4 males (mean 30 years) also performed the odor detection test. The study was approved by the Regional Ethical Review Board in Stockholm and performed after informed written and oral consent (Dnr 2016/1229-31/4 and Dnr 2016/2556-22).

### 2.3. Odor Perception and Description

An odor threshold around 5 ng/m^3^ of 3CA has been suggested. Our pilot experiment with three healthy volunteers showed that 50 ng/m^3^ of 3CA was detected by all and 3.1 ng/m^3^ by none [18]. At first visit, participants were therefore made familiar with the odor-bag testing system by first smelling from one bag filled with 3CA at 50 ng/m^3^ and then one with clean air. All participants perceived a difference between the odor-bags. Participants evaluated their sniff from the 3CA odor-bag using a Borg scale [20] ranging from 0 representing “no percept at all” to 120 representing “absolute maximum”. The following endpoints were used to give an odor description: (1) Odor intensity, (2) odor un/pleasantness, (3) dry mucus, (4) itching, (5) throat irritation, (6) breathing difficulty, (7) concentration difficulty, (8) dizziness, (9) tiredness, (10) headache, and (11) nausea; and the following characteristic adjectives were given to describe the odor perception: (12) moldy, (13) musty, (14) stuffy, and (15–17) up to three own odor quality descriptors. Endpoint 2 (un/pleasantness) was rated from −120 to +120, where negative ratings are interpreted as unpleasantness.

### 2.4. Odor Detection

**Figure 1:**
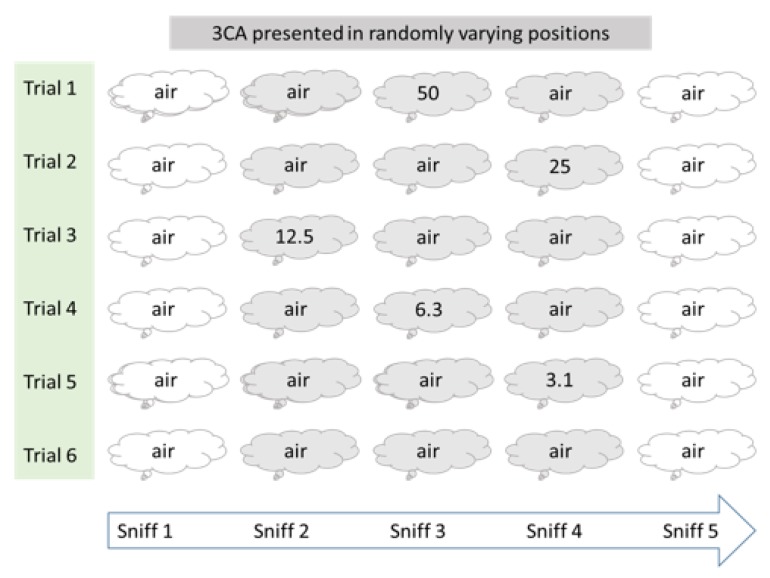
Oder detection setup. The presentation of 2,4,6-trichloroanisole (3CA) at different concentrations (ng/m^3^) was randomly assigned to the 2nd, 3rd, or 4th sniff.

The presentation of odor-bags was done in a single-blinded manner, with participants using blindfolds during trials in a concentration interval from 50 to 0 ng/m^3^ of 3CA. The test was organized in six successive trials with descending concentrations in the first five trials (50, 25, 12.5, 6.1, 3.1) and zero concentration in the sixth trial (0, sham). Each trial comprised five sniffs, the first always from a bag with clean air, followed by a triplet of bags in random order where two contained clean air and one contained 3CA, and finally a fifth bag with clean air (Figure 1).

Participants were informed that each trial started with a clean air bag and instructed to point out the bag with 3CA as soon as they perceived it, upon which the individual had to rate their confidence (“sure” or “not sure”). If no bag was identified during a trial, despite the instruction, the participants had to give a forced-choice identification and confidence rating (“followed a feeling” or “random answer”). After forced-choice or wrong identification, participants were given to sniff the current bag with 3CA again. A ten-minute rest was given between trials, with decreasing concentrations: 50, 25, 12.5, 6.3, 3.1, and 0 ng/m^3^. All participants performed all trials, but results are only given until participants failed. Correctness of answers (coding yes, no) are reported, as well as confidence ratings for all correct answers (coding 0–3, as follows: 0 = “random”, 1 = ”followed a feeling”, 2 = ”not sure”, 3 = “sure”).

### 2.5. Statistics

Descriptive statistics were used to show performance for 3CA odor perception, description and detection. Due to large spread and inter-individual variation, medians rather than averages are presented. The relation between perceived odor pleasantness and tendency to report odor-related symptoms was analyzed by Spearman rank correlation. Data from odor detection are analyzed as success rate in percent on group level and mean of corresponding confidence ratings. Only correct trials until first mistake were considered. Statistics, tables and figures were done using R and Microsoft Excel.

### 2.6. Historical Investigation

Key information from Swedish texts is presented as translated citations (tr), in plain text, or italics for titles. Information search was guided by authors’ prior knowledge and personal communications following previous publications, mainly the E-publication of our Indoor Air paper [2], which received attention in media and among practitioners, scientists and authority officials. A senior expert on moisture in buildings at the Swedish Agency for Housing Building and Planning, revealed a historic link between house foundations and CPs, which is described in a national journal [21]. The governmental retraction of licenses for CPs was retrieved from a website of a company performing building remediation (www.lfs-web.se). A private person contributed with pictures for publication on the building of a house from ÅsedaHus. Information was also retrieved from the world-wide web, authorities, the library of the Occupational and Environmental Medicine clinic in Stockholm, and from the National Library of Sweden including the digitalized newspaper archive. Going back to the early 20th century, information was searched in books, journals, governmental reports, and newspapers, and was guided by, but not limited to, Swedish words for buildings, indoor, odor, nasty odor, mold odor, mold, dampness, wood preservatives, wood rot, wood decay fungi, CPs, CAs, various adverse health effects and names of people, institutions, companies, and products linked to retrieved results.

## 3. Results

### 3.1. Olfaction Experiments

#### 3.1.1. Odor Perception of 3CA

The Borg scale ratings of intensity were rather low for the 44 participants (Table 1), median 18.5 (weak to moderate). Un/pleasantness of 3CA odor was rated as “negative” by 25 participants, “neutral” by 7 and “positive” by 12, with individuals perceiving 3CA as less pleasant tending also to report it as more intense (Spearman rho = −0.29, *p* = 0.05), this being a well-known relationship for many odorants. Furthermore, data showed large inter-individual variation with intensity ratings ranging from 1 (nothing to extremely weak) to 65 (strong to very strong). Inter-individual variability was also seen when occurrence of nine chemosensory related symptoms was rated, resulting in median values of 0 but with individual maximum ratings up to 70 (very strong) for concentration difficulty, and 50 (strong) for nausea. Interestingly, individual tendency to report any chemosensory related symptom (sum of symptoms) aligned with ratings of perceived 3CA unpleasantness (Spearman rho = −0.3, *p* = 0.044) but not intensity (Spearman rho = 0.25, *p* = 0.1). However, these relationships need further investigation because of the low number of individuals reporting more than one chemosensory related symptom at 50 ng/m^3^ (Table 1).

#### 3.1.2. Odor description of 3CA

The Borg scale ratings of verbal descriptors “moldy”, “musty/stale”, and “close/stuffy“ were weak within the group of 44 participants (Table 1), with median ratings of 0, 3, and 6 respectively. However, some participants rated the descriptors as 120 = absolute maximum, demonstrating large variability between participants. Furthermore, participants were allowed to name up to three additional odor quality descriptors, which afterwards were grouped as: Chemical (count = 5, median rating = 40, range = 3–95), house-related (14, 45, 3–120), chlorine (5, 25, 3–50), plastic (4, 21.5, 10–35), rubber (2, 55, 25–85), and other (5, 15, 3–70). Interestingly, house-related descriptors (basement, old house, stuffy house, moist house, moldy house) were by far the most commonly chosen odorant-descriptors (*N* = 14) even at the group level, as indicated by a median rating of 45 (strong). Of these 14 individuals, six rated the odorant as unpleasant (median = −17.5, weak to moderate unpleasantness) and six as pleasant (median = 22.5, moderate pleasantness), showing that 3CA can have both positive (summerhouse, holiday) and negative associations (stuffy, moist, moldy).

#### 3.1.3. Odor Detection of 3CA

**Figure 2:**
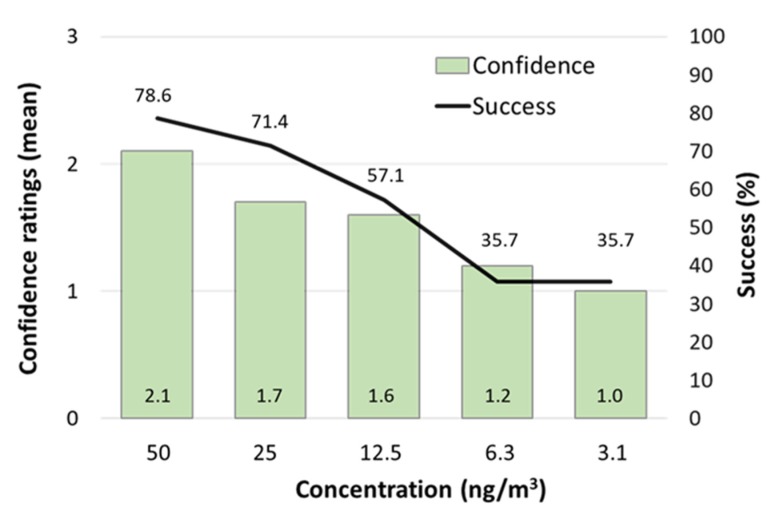
Odor detection of 2,4,6-trichloroanisole (3CA) among 14 healthy volunteers. The solid line connects the percentage of subjects that identified 3CA at different concentrations. The bars show the mean level of confidence among the subjects that identified 3CA (coding 0–3, as follows: 0 = “random”, 1 = ”followed a feeling”, 2 = ”not sure”, 3 = “sure”).

Of the 44 consecutive study participants, the last 14 were tested in the concentration range from 50 to 0 ng/m^3^ (Figure 2). Three of four males failed at 50 ng/m^3^, even though they had previously recognized odor at this concentration. Still, more than two thirds of the 14 participants successfully detected 3CA at 50 and 25 ng/m^3^ (79% and 71%, respectively), more than half detected 3CA at 12.5 ng/m^3^ (57%) and more than one third (36%) even detected 3CA at the two lowest concentrations (6.3 and 3.1 ng/m^3^). Taking a closer look at confidence ratings given by participants after successful selection of odor bag (results not shown), we see that the highest confidence rating (“sure”) was only used by half of the participants (50%) and only after sniffing 3CA at the three higher concentrations (50, 25, 12.5 ng/m^3^). When participants in the last trial had only bags with clean air to choose from, but were told that one contained 3CA, all but one participant (93%) gave low confidence ratings (36% “followed a feeling”, 57% “random”).

### 3.2. Historical Investigation

#### 3.2.1. Reasons Why CPs Were Promoted in Building Constructions and Evidence of Indoor Use

**Figure 3:**
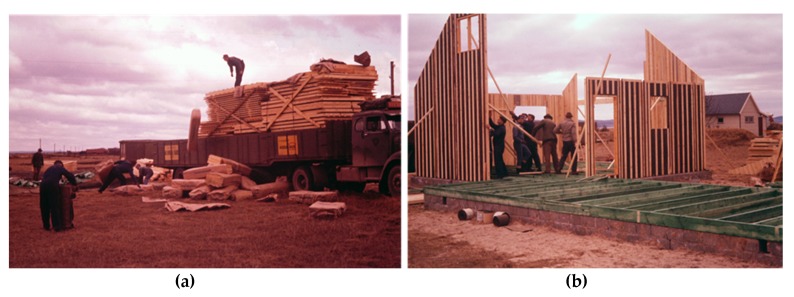
(**a**) Delivery in the early 1960s of a house from ÅsedaHus, one of many companies that produced prefabricated homes in the 1960s and 1970s [32], as well as, e.g., kindergartens, schools and offices. (**b**) The house is erected on a ventilated crawlspace with preserved wood (green) that will eventually be invisible below the interior flooring. ÅsedaHus used KP-Cuprinol for impregnation and chlorophenols (CPs) for dipping, as reported by the company [33] and supported by historical investigation before sampling of soil [34] and analyses of polluted soils showing CPs and dioxins at the manufacturing site [35].

Already in 1927, experts from the National Testing Institute (SP) concluded in the report *“Serpula lacrymans and conservation of wood against rot”* (tr) that it was of national economic interest to protect buildings against wood decay fungi [22]. SP became responsible for approving wood preservatives for dipping, brushing and spraying while chemicals for impregnation were approved by the Swedish Wood Protection Committee/Institute (STSK/I). Approvals were not related to health, being solely based on efficacy against rot. In 1950 the first “Material and workmanship guide” for best practices in house building stated that “unless otherwise specified, timber in contact with foundations walls should be conserved” (tr) [23]. At this time, such timber was also protected by traditional moisture barriers. However, a direct need for preservatives emerged with the introduction in a manual from 1954 of ventilated crawlspaces (Figure 3) and concrete slabs on the ground, mainly inspired by the developments in the U.S.A according to the references [24,25,26]. These designs without basement had inherent problems with moisture and were prone to attack by wood decay fungi [27]. To avoid rot and risk of house collapse, institutions and authorities increasingly promoted wood preservatives applied by different methods in many parts of buildings. Bönnelyche & Thuröe (BT), the company producing KP-Cuprinol, even participated in work to update the “Material and workmanship guide” regarding wood preservation, together with five other Swedish companies. Three of the companies, i.e., BT, Boliden and Höganäs (that later acquired BT), published in advance the committees´ suggestions of vastly expanded preservative usage [28]. In 1970, impregnation was even recommended in a national building code comment as an alternative to physical moisture barriers (1970/3 SBN32:241) [29], thus allowing impregnated timber in direct contact with foundations that are often damp in temperate climates. The two major products used for impregnation were Swedish and originally patented by the native inventor Bror Häger, who received a gold medal from The Royal Swedish Academy of Engineering Science in 1961 for his work on preservatives [30], i.e., K33 (chromated copper arsenate, CCA, from Boliden), and KP-Cuprinol (copper and CPs, from BT). A third product called BP-Hylosan (oil and PCP) was less used but, as advertised by Swedish British Petroleum [31], it was used by leading producers of timber and prefabricated homes, e.g., Mo och Domsjö and Hulfredshus, respectively.

**Figure 4:**
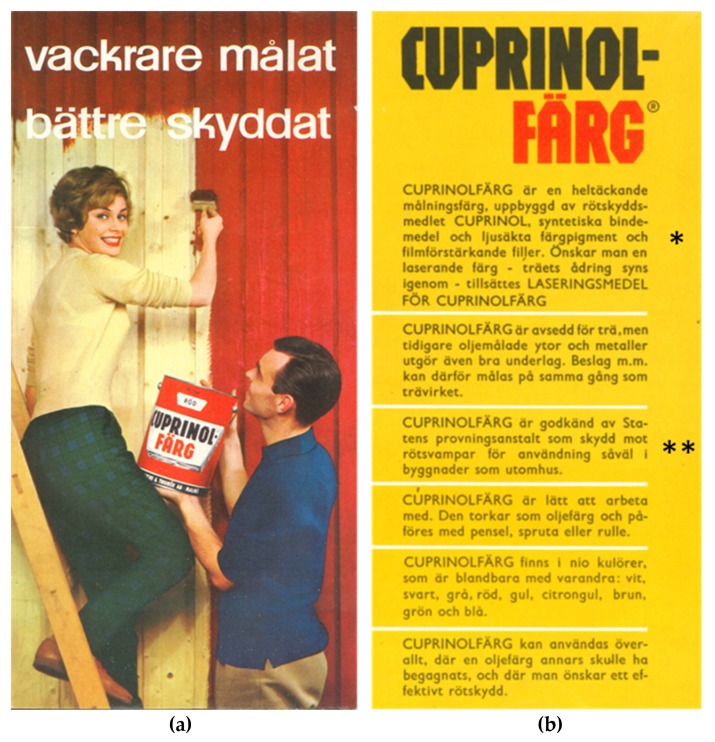
(**a**) Front page of an advertisement pamphlet *“More beautifully painted, better protected”* (tr), issued by Bönnelyche & Thuröe AB, dated 9.3.1960. (the company does not exist today [45]). (**b**) Content informs that *“CUPRINOL PAINT is made of the preservative CUPRINOL” (tr) and **“approved by the National Testing Institute for protection against wood decay fungi for use both in buildings and outdoors” (tr).

Consumers were made well aware of CPs when first marketed around 1954, as they were emphasized in advertisements, e.g., for BP-Hylosan [31] “the strongest protection against all that attack wood” (tr), and for Cuprinol paints [36,37] “PCP, the most effective rot protection agent known to science” (tr). CP-based products were approved by SP for use indoors and outdoors, which was highlighted in advertisements, e.g., for Cuprinol paint (Figure 4) [38] (see the 1960 list of products in: Appendix A, Table A1 [39]).

#### 3.2.2. Reasons Why CPs/CAs Were Overlooked

● Public unawareness

With time, CPs were not mentioned in advertisements and the popular Cuprinol trademark became synonymous with anti-rot function (Figure 4) [38]. It was also not obvious to consumers that CPs were present in impregnated wood as it was generally hidden, being built into constructions e.g., in the contact between house and foundations such as crawlspaces (Figure 3). In addition, many CP-based products were colorless and not clearly visible.

● Unnoticed withdrawal of licenses for CP-based products

An authority decision from 1977 retracted all licenses for CP-based products at the end of the year, except BP-Hylosan which was allowed also during 1978 [40] (see all products and producers listed in: Appendix A, Table A2). The process leading to the decision was described as rapid and contained the sentence “to avoid an inevitable and improper discussion in media” (tr). Directors of several major governmental agencies were involved, and the process included STSK/I and SP. A supplement to the decision described Swedish research on CPs and dioxins, and knowledge of severe adverse health effects, including fatalities of piglets and children. Many vendors of CPs were active in other industrial sectors than wood conservation, because the biocides were added to paints and petrochemicals such as mineral spirits and oils. Many of the 56 withdrawn products were used in house constructions and homes, but media reported on the decision as being merely of occupational relevance. Following the withdrawal, many consumers and builders were probably unaware of the changes in the compositions of well-known labels, such as Cuprinol. Moreover, CPs left few traces in later documents. When STSK/I presented historical amounts of impregnated wood in 1984, it was neither possible to see trade names, nor the past use of CPs, due to changed categories of product compositions [41]. It seemed as if CPs had never occurred indoors in Sweden, e.g., in a large newspaper article covering a court case in Western Germany, *“Did the children die of the dangerous paint?”* (tr) [42]. Furthermore, two reports from SP stated, in a discussion on indoor air CPs in Western Germany, that “beside volatiles from creosote, no cases of active preservatives have been reported in the Nordic countries” [43,44].

● Nasty odor was attributed to mold instead of impregnated wood or CPs.

An early example from 1973 is captured by two newspaper articles and headlines on the same case, first *“The school stinks—but nobody knows why”* (tr) [46], where the appointed building investigator suspects impregnated wood in the floors and facing of a new prefabricated school, and then *“Mold—that is why the school stinks”* (tr) [47], where mold is blamed but the information is given that “experts expected the impregnated wood to be affected by rot but it was fresh” (tr). A report from the National Swedish Institute for Building Research in 1974 is dedicated entirely to the nasty odor described in newspapers, sometimes pointing to impregnated wood. The report contained an odor experiment with three pieces of wood, one non-treated, and two impregnated with either CCA or KP-Cuprinol. The materials were placed in separate jars and kept moist. After eight months, nasty odor similar as in affected houses was emitted from the three pieces. The report concluded that impregnated wood was not important for the development of nasty odor [48]. Moreover, the author added a comment in a reprint from 1979 that the odor is caused by a certain type of mold and concluded that the main problem with all affected buildings was deficits in protection against dampness [49]. In 1982, authors from SP and STSK/I stated that “impregnated wood protects against rot but not against mold” (tr), in the report *“Dampness and mold”* (tr) [50]. This title expression rapidly became common language. A national “Dampness and Mold Fund” (tr) was established, to provide financial help to affected house-owners, provided constructional deficits could be documented by building investigators. A presentation by the fund in 1988, shows that nasty odor was the major problem [51], and the most common damages corresponded to those that typically contained preservatives, e.g., wood in direct contact with concrete in foundations. SP was a key player in the investigations of odorous buildings, including major cases involving civil litigations concerning nasty odor in large groups of completely new houses. One such case was included in a report that pointed to impregnated wood and stated that “fungi that attack impregnated wood often cause severe odor” (tr) [52]. We retrieved a description by SP of a typical problematic house, as presented to the public in two reports from 1981 and 1985, i.e., *“Houses with mold odor”* (tr) [53] and *“Mold in houses”* (tr) [54], respectively. As described, mold odor was a new phenomenon in buildings that often occurred along skirting boards, but it was typically difficult to find visible dampness and mold, even when opening floor constructions. The reports illuminate the long-lasting costs and frustration for people that do not know the reasons for odor formation (see Appendix B: Original description of odorous houses, by SP).

● Circumstances pointing to impregnated wood or pointing away from mold were disregarded

Already in 1980, a Swedish toxicological review described CPs/CAs in soils and as cause of odor or taint in the broiler industry [55]. A document from STSK/I relates to the paradoxical lack of mold in houses affected by odor. Due to lingering suspicions of impregnated wood as a source of odor, and risk of a general ban against wood preservatives in the built environment, STSK/I performed a separate investigation. The resulting 1994 report, *“Nasty odor from impregnated wood”* (tr), pointed to the already banned KP-Cuprinol [56]. Furthermore, the previous claim that odor was due to mold growth was retracted, as it was sometimes difficult to see growth on odorous pieces of impregnated wood, even under a microscope. The report even stated that odorous constructions rarely contained mold [56]. SP also concluded that “the odor of impregnated wood can be confused with mold” (tr), in a report from 1998 on *“220 schools, damage and defects in school buildings”* (tr) [57]. Laboratory analyzes of mold odor due to CPs/CAs hit the market in 1999 [58] and were presented in 2000 at a Healthy Buildings conference [59]. A few years later, methods and results were also published in a scientific paper by German researchers studying indoor musty odor [1]. In 2009, the Swedish Environmental Protection Agency mentioned KP-Cuprinol as a source of mold odor [60].

● Indoor air researchers attributed odor and health effects to mold only

In the late 1970s, newspapers reported on symptoms linked to mold odor, e.g., headache, asthma, and allergy [61,62]. At the Indoor Air conference in Stockholm in 1984, SP described a completely new building defect [63], “during the past ten years an increasing number of sick houses have been reported in Sweden, which suffer from mold odor due to the growth of mold within the structure”. Furthermore, occupants were “troubled by an offensive odor, and suffer from various degrees of medical complaints”. Moisture and basementless constructions were highlighted as causative factors, and it was emphasized that “damage to buildings due to rot had declined but had been replaced by the problem of sick houses” [63]. The same year, the Swedish National Board of Health and Welfare claimed that geosmin produced by actinomycetes explained the mold odor [64], but that claim was not repeated, to our knowledge. The medical complaints engaged staff at Swedish occupational and environmental medicine (OEM) clinics. Tri-annual indoor environment conferences began in 1985, and research was performed at OEM clinics connected to universities. In 1989, a supplement to the Swedish Government Official Report “The Allergy Investigation” (tr) [65], stated that odor was typical of a sick building. Mold was a suspected factor underlying increase of allergy and hypersensitivities, and support to research and epidemiological studies was called for by the experts. Many epidemiological publications followed. One study reported association of recurrent wheezing in children with living in private homes with crawlspaces and concrete slabs on the ground [66]. Another study reported association between allergic symptoms among children and moldy odor along the skirting board [67]. It was discussed that foundations are often problematic and that “microbiological or chemical degradation products from affected building material from these parts of the construction may play a role both for odor problems and studied health effects” [67].

## 4. Discussion

We present experimental psychophysical data combined with a historic chain of events that is far from complete but sufficiently detailed to advance knowledge on the CAs/CPs in the built environment.

Our historical investigation uncovers and transfers key Swedish information to the English-speaking scientific domain and provides approaches, e.g., building regulations and advertisements, that researchers may use to reveal similar applications of CPs in other countries. An additional approach, not used here, might be to scrutinize national regulations on hazardous building waste.

Regarding the question why Swedish institutions promoted preservatives in buildings, we identify one key reason, namely the import from other western countries in the early 1950s of new designs of house foundations without basements. In practice these designs of crawlspaces and concrete slabs on the ground were prone to moisture and rot. However, chemical methods to prevent rot in outdoor wooden posts, poles and railway ties were already at hand [2] and chemical industries contributed with both products and best practices in response to a rising demand from societal sectors involved in building and planning. As a result, the foundation designs were expanded to include wood preservatives, while efforts were made to understand and solve their inherent problems with dampness. With this chain of events, biotransformation of CPs to CAs is a natural consequence. It seems telling that the National Swedish Committee/Institute for Building Research issued the first of three manuals on *“Houses without basements—with special reference to foundations”* (tr) in 1954, followed by a report on *“Moisture and rot damages in wood houses without basements”* (tr) in 1964, and then in 1974 the first of two reports on *“Nasty odor in houses without basements”* (tr) [24,25,26,27,48,49]. Beside the problem with odor, our investigation uncovers a considerable indoor use of and exposure to CPs in Sweden, and we list many used products, including the Swedish Cuprinol that became so popular in the 1960s that people said “to cuprinole” instead of “to paint” [68].

In line with this historical account, similar events appear to have occurred a few years earlier in the USA where PCP was promoted to the American public already in 1953, to treat wood in crawlspaces, and many other locations in buildings, and for indoor applications [69], leading to general population exposure [70]. Most likely, CPs were similarly used in many other countries, it is therefore notable that CAs have so far only been reported indoors from one country besides Sweden. In Germany, CAs are recognized as a cause of “musty” odor [1,71], often called Geruch in Fertighäuser (“odor in prefabricated houses”), which is described on many websites as being mold-like, and continues to appear in scientific publications, see e.g., [72].

When investigating in Sweden if present day levels of CAs are detectable by olfaction, we included rating of confidence, so that participants should not only choose the correct bag with 3CA but also rate their confidence in the choice [73]. The presented concentrations are likely to be equal to or lower than intended. When using this method to present descending concentrations of 3CA, we observed that most participants could detect the odorant down to 13 ng/m^3^, in several cases with high confidence. Five out of 14 participants also made no mistake at 6.2 and 3.1 ng/m^3^, albeit with less confidence.

Our experiments were not designed to determine an odor threshold, however, they demonstrate detectability below 5 ng/m^3^ previously reported as an odor threshold of 3CA in a conference paper [17]. Concerning our question whether the low concentrations of 3CA reported in indoor air can be perceived, the answer is yes, since our test concentrations were within the range reported in buildings with odor complaints [2]. Furthermore, the large inter-individual variability in our study suggests a risk for nuisance among some but not all people living in 3CA burdened homes. Today, concentrations of CAs are reported to be in the low ng/m^3^ range, just as for CPs. Levels were likely much higher in the past, around 50 µg/m^3^ have been reported for PCP from several countries [74,75], and PCP is the least volatile of all CPs. Levels of many other indoor chemicals have also decreased over time [70].

Concerning the question if the odor of 3CA can be described as moldy, the answer is also yes, although the volunteers in our study gave variable answers. Thus, 15 participants chose moldy as a descriptor, 26 chose musty, and 30 chose stuffy. Furthermore, when participants were asked to name their own odor qualities, the variation increased and included descriptors such as chemical and chlorine. Still, house-related descriptors were most common but not all participants that used such descriptors considered the odor unpleasant. Maybe some participants have positive memories associated with the odor, such as vacation as a child in a summerhouse with treated timber. It should be noted that young participants were used, the results might be different with affected homeowners, building inspectors or people with experiences from the 1960–1970s when CA levels were most likely much higher than today. Furthermore, the odor quality may be affected by combinations of CA congeners, which in turn depends on the ratios and types of CPs used in a specific building, usually 2,4,6triCP, 2,3,4,6-tetraCP and PCP.

We conclude that current low concentrations of CPs in problem houses are still relevant via odor perception. We further present a chain of evidence demonstrating that the nasty indoor odor that emerged in large scale in the 1960–1970s was due to a shift in building practices, from protecting wood from dampness, to accepting dampness in foundations and instead protecting wood from rot using “rot protection agents” (as these biocides are called in Sweden). However, we argue that the national authorities involved in this shift established a narrative of “dampness and mold” instead of “dampness and rot protection agents”. We argue this “dampness and mold” narrative to be the overall reason for the unawareness of peoples´ exposure to CPs/CAs, not only among the general Swedish population but also among researchers in Sweden and elsewhere. It is evident that nasty odor was also mold odor, and CAs are indeed produced by microbes but only in the presence of CPs. Nonetheless, as shown here, mold became the singular source of mold odor recognized by governmental agencies after the withdrawal of CPs in 1977–1978, creating a gradually increasing dichotomy with practitioners that continued to point to impregnated wood and embraced analyses of CPs/CAs in air and building materials already in 1999.

Notably, we demonstrate that industry promoted CPs and even participated in preparing a “material and workmanship guide” for building houses, and that at least one of the two institutions that registered “rot protection agents” also had ties to industry, i.e., STSK/I. The other institution, SP, was a national agency (1920–1993) and later a national company (1993–2017) that sold building investigations, chemical and microbial analyses, and had experts that co-authored scientific articles, including epidemiological studies and reviews. It cannot be excluded that some of their reports and studies were designed and written by authors well-aware of but not mentioning impregnated wood and CPs/CAs. The Swedish neglect of CPs described herein may very well have influenced the evolving indoor air research where Sweden and Denmark had leading roles [76,77,78].

We presently investigate early developments in other countries and international organizations but it is seems appropriate to mention here that the first report from the WHO working group on indoor environments in 1979 presents German examples of PCP in homes and highlight only three other hazards in building materials, namely radon, asbestos and formaldehyde [79]. Thereafter, indoor exposure to CPs was considered in other contexts, such as specific toxicological reviews e.g., by the International Programme on Chemial Safety [74,75].

The second WHO report from 1983 includes Swedish examples and introduces the concept of “sick-building syndrome” [80]. By then, Swedish newspapers had already reported on a link between mold odor, sick buildings, and acute airway symptoms. It therefore seems likely that CPs/CAs incited and provided impetus to the Swedish epidemiological research carried out up to and even past the WHO report on “Dampness and mold” in 2009 [3]. In fact, already in 1981 the chairman of the National association against asthma and allergy stated in a newspaper article that the increase of allergy coincided with the “one million homes program” completed in the 1970s, and was due to indoor factors such as “dampness and mold”, formaldehyde, glue, and chemicals in paints [81].

It is not the scope of this paper to review Swedish scientific literature on “dampness and mold”, but it seems pertinent to discuss some issues relating to our results that CPs/CAs were confused with mold. Firstly, in the 1970s, the odor problems coincided with significantly higher exposures to hazardous chemicals including CPs. Secondly, the early epidemiological studies were performed well after public alarm about odor in the 1970s. Therefore, the associations found may be due to other exposures than “dampness and mold” and/or may include psychobiological effects and methodological weaknesses since answers to questionnaires may reflect preconceived beliefs. As an example, an early Nordic review from 2001 concludes that “the evidence for a causal association between ‘‘dampness’’ and health effects is strong” [82]. The authors discuss different types of bias and consider it improbable that recall bias is a major explanation for associations, because “some authors note that the associations were observed already in the late 1980s – at a time when the general public was not aware of a possible association between “dampness” and health effects”. The authors did not specify which countries they had in mind, but at least the Swedish population was very well-aware and Swedish studies may therefore be biased.

In fact, public awareness increased even more following the Nordic review, as did public health actions against “dampness and mold”. One step was a suggestion in 2002 originating from the OEM clinic in Stockholm, that mold odor, visible mold and visible dampness should be used in a governmental national environment program as indicators of hazardous exposure and included in regular nationwide questionnaires [83]. Every fourth year since then, these indicators have been highlighted in the national environmental health reports as major risk-factors for asthma, allergy and sick building syndrome type of symptoms. According to the last report, around 20% of the Swedish population is allergic or hypersensitive to mold, and around 20% is exposed to dampness and mold [84]. These national reports receive media coverage and give the impression that Swedish residential indoor air often contains high levels of hazardous substances. Furthermore, the national reports are followed-up by regional reports from several OEM clinics generating additional media attention. It is possible that all this alarm around mold leads to problems, including increased environmental sensitivity, attribution of symptoms, and development of symptoms through mechanisms such as nocebo. We are currently investigating how negative information bias affects the perception of CAs in people with and without multiple chemical sensitivity (MCS).

Concerning mold, we present evidence to suggest that substantial microbial growth was not required for development of odor in buildings from the 1960s and 1970s. Rather, it seems that minute microbial activity over time produces CAs in sufficiently high concentrations to evoke odor. In any case, odor may be a problem for those exposed, and it does not matter if it is perceived as nasty, moldy, musty, bad smell, smell or just odor. Still, it is important to balance the information to the public on exposure and risk of adverse health effects and we see a need for critical evaluation of epidemiological studies on “dampness and mold” concerning their validity for people today [85].

Obviously, information to the public is crucial for rational and cost-effective remediation efforts of CPs/CAs in any country and it now seems that Swedish institutions start to recognize CAs as a cause of mold odor, e.g., in the latest National Environmental Health Report from The Public Health Agency of Sweden [84], and in an information booklet on indoor environment in schools from The Swedish Association of Local Authorities and Regions [86]. One national journal article even contains a statement by an indoor environmental specialist in Gothenburg that CAs continue to be a major cause of problems in buildings from the 1960s to 1970s [21]. The referred specialist oversees buildings administered by the City of Gothenburg, one of the largest property managements in Sweden, taking care of more than 3000 buildings with around 150,000 residents in e.g., pre-schools, schools, offices, and homes for elderly.

## 5. Conclusions

Swedish institutions introduced building foundations with inherent dampness problems that lead to rot. They countered rot with CPs, approved CPs as household chemicals, and then banned CPs without people being fully aware of their presence in buildings. They attributed odor and health effects to “mold”, and established a narrative of “dampness and mold” that disregarded CAs, even though these chemicals seem to be the only volatiles linked to odor in buildings nationwide. Today, CPs in buildings still give rise to CAs that continue to be an indoor environment problem, since the low concentrations can still evoke unpleasant moldy odor as shown here. We suggest that the research on “dampness and mold” may have been confounded by focusing on improper exposure factors, thus warranting a re-evaluation of the research in this area. We argue that this is in important issue, as people have a right to know about their exposures in daily life and the associated health risks—or absence thereof.

## Figures and Tables

**Table 1:** Individual ratings by 44 subjects sniffing from a bag containing 50 ng/m^3^ of 2,4,6-trichloroanisole. The ratings were given on a Borg scale ranging from 0 (no percept at all) to 120 (absolute maximum). Ratings around 10 are labeled as “weak”, ratings around 25 as “moderate”. The ratings of “Unpleasant” are assigned negative values to allow comparisons with those of “Pleasant”.

Rating	Count	Range	Median	25th Percentile	75th Percentile
Intensity	44	1 to 65	18.5	10	25
Females	31	2 to 65	15	10	25
Males	13	1 to 57	20	10	33
Un/Pleasantness	44	−90 to 100	−2	−5	3.25
Unpleasant	25	−90 to −1	−5	−2	−15
Pleasant	12	2 to 100	23	14.5	30
Neutral	7	0	0	0	0
Chemosensory symptoms					
All ratings	44	0 to 8	1	0	1.25
>1 symptoms ^†^	11	2 to 8	3	2	5
Odor qualities:	44				
Mold smell	44	0 to 120	0	0	2
Musty smell	44	0 to 120	3	0	17.5
Stuffy smell	44	0 to 120	6	0	25

^†^ Subgroup of participants responding with one or more symptoms to 2,4,6-trichloroanisole.

## Appendix A

**Table A1:** Products with preservatives that were approved for use in buildings and outdoors by Statens Provningsanstalt (SP) in 1960. The products were intended for brushing, dipping and spraying.

Company	Product	Origin ^†^
AB Bönnelyche & Thuröe, Malmö ^‡^	Cuprinol, färglös, ljusbrun, brun, grön	SE
AB Bönnelyche & Thuröe, Malmö ^‡^	Cuprinolfärg, brun, grön, gul, blå, röd,	SE
	svart, vit, grå, citrongul	SE
Höganäs-Billesholms AB, Höganäs ^‡^	Håbinol, färglös, grön, brun	SE
Höganäs-Billesholms AB, Höganäs ^‡^	Håbinolfärg, brun, tegelröd, röd, grön, gul,	SE
	vit, blå, grå, svart	SE
Höganäs-Billesholms AB, Höganäs ^‡^	Håbisan, färglös, brun	SE
Svenska BP Oljeaktiebolaget, Stockholm ^‡^	BP-Hylosan	GB
Svenska BP Oljeaktiebolaget, Stockholm ^‡^	BP Hylosan G1	GB
Ing.firma Pehr Engwall AB, Stockholm ^‡^	Solignum nr 25 VDK	DK
Ing.firma Pehr Engwall AB, Stockholm ^‡^	Solignum nr 2, mellanbrun	DK
Condorverken AB, Göteborg	Xylamon-Natur	DE
AB Joh. Ohlssons Tekn. Fabr., Stockholm	Carlinum nr 1, ljusbrun	-
AB Oljerafinaderiet Ceres, Malmö	Coppersat, grön	-
Tekn. Lab., Bror G. Hellström, Göteborg	Cupsolat kopparolja	-
Evers & Co -AB, Hälsingborg	Evrisan, färglös PF, färglös YF, brun, grön	-
Evers & Co -AB, Hälsingborg	Evrisanfärg, brun, grön, gul, blå, röd, svart,	-
	vit, grå	-
AB Joh. Ohlssons Tekn. Fabr., Stockholm	Kopralin, grön	-
AB Zonen, Malmö	Kupferit: brun, grön	-
Sv. Oljekonsumenters Riksförbund, Stockholm	0. K. Träimpregneringsmedel, brun	-
Sv. Oljekonsumenters Riksförbund, Stockholm	0. K. Kopparolja, grön	-
Engqvist & Udesen, Göteborg	Patinol, grön	-
Skogens Kol AB, Kilafors	Preservol, brun	-
AB Oskar Dahlin, Stockholm	Rustikol, brun	-
AB Rötmotaverken, Korsnäs	Rötmota Export, brun	SE
Fabriks AB Fernisol, Malmö	Timravol, grön	-
Fabriks AB Fernisol, Malmö	Timravol, brun	-
AB Mataki, Malmö	Transote, brun, grön	-
Firma Ernst Lennström, Göteborg	Örnolit, brun, grön	-

Data on companies and products from Statens Provningsanstalt, Cirkulär 22, August 1960. ^†^ Origin (country) of product or label to the best of the authors´ knowledge, “-” means unknown (in most cases SE). ^‡^ Producer or provider of products with chlorophenols (CPs) around 1960, although not all products may have contained CPs, and products of unmarked companies may have contained CPs.

**Table A2:** Products that had their licenses withdrawn by Produktkontrollnämnden in 1977. The products contained chlorophenols (CPs) and were intended for fabrics, paints, wood preservation and more.

Company	Product	CPs ^†^	License	Origin ^‡^
BT Kemi KVK AB ^§^	BT Blåskydd	TriCP	2948	SE
Höganäs Färg AB	KP-Cuprinol ^¶^	PCP	3053	SE
Höganäs Färg AB	Zink Cuprinol färglös II	PCP	2394	SE
Höganäs Färg AB	Koppar Cuprinol Grön 10	PCP	2395	SE
Höganäs Färg AB	Husbockscuprinol	PCP	2431	SE
Svenska BP AB	BP-Hylosan ^¶^	PCP	3055	GB
Svenska BP AB	BP-Hylosan Kulör	PCP	3197	GB
Svenska BP AB	BP-Hylosan WR	PCP	3198	GB
Svenska BP AB	BP-Hylosan BF 55	PCP	3199	GB
Monsanto Scandinavia AB	Santobrite	PCP	2430	US
Dow Chemical AB	Dowicide G	PCP	3097	US
Sikema AB	Witophen N	PCP	3100	DE
Svenska Rhodia AB	Xylophene NA	PCP	2429	FR
AB Sadolins Färgfabrik	Pinotex	PCP	2814	DK
AB Sadolins Färgfabrik	Sadolin Sadovac	PCP	3238	DK
AB Sadolins Färgfabrik	Grundtex	PCP	3265	DK
AB Sadolins Färgfabrik	Toptex	PCP	3301	DK
Gori AB	Gori Grund	PCP	2939	DK
Gori AB	Gori 44	PCP	2947	DK
Gori AB	Gori 66	PCP	2948	DK
Ingenjörsfirma Pehr Engvall AB	Penta-Solignum	PCP	2703	DK
Ingenjörsfirma Pehr Engvall AB	Kreosit-Solignum	PCP	2705	DK
Gullviks Fabriks AB	Gullviks Husbocks-Cupral	PCP	2576	SE
Gullviks Fabriks AB	Servarex Granulat	PCP	3094	SE
Gullviks Fabriks AB	Gullviks Blåskydd	TriCP	3024	SE
Gullviks Fabriks AB	Servarex Teknisk	TriCP	3093	SE
Gullviks Fabriks AB	Servarex Teknisk	TetraCP	3093	SE
Gullviks Fabriks AB	Servarex Teknisk	PCP	3093	SE
AB Engqvist & Udesen	Penta-Cuprileum	PCP	2577	-
AB Henning Persson	Triconol ljusbrun	PCP	2334	-
AB Henning Persson	Triconol mörkbrun	PCP	2335	-
AB Henning Persson	Treepinol Kopparolja grön N	PCP	2337	-
AB Henning Persson	Triconol Kopparolja grön	PCP	2338	-
AB N Haglund Färgindustri	Master Patent	PCP	2969	-
AB Plantex	Bocan C Extra	PCP	3047	-
AB Plantex	Penta-NA	PCP	3095	-
AB Plantex	Plantex Tri-K	TriCP	2112	-
AB Pulco	Pulco Fenolat	TriCP	3104	-
AB Rötmotaverken	Penta-Fiberol 1142-F Färglös	PCP	2620	SE
AB Rötmotaverken	Penta-Röanol 1105-1235, brun	PCP	2621	SE
AB Rötmotaverken	Penta-Rötmota PKF 1106, brun	PCP	2573	SE
AB Svensk Färgkemi	Färmi-Penta	PCP	2735	-
AB Svenska Byggnadsisolering	Penta-Copperol brun	PCP	2729	-
Anti Pa AB	Anti-PA iV Husbock	PCP	2585	-
Anticimex AB	Gantix HB ljus	PCP	3065	-
Anticimex AB	Anthyllit M	PCP	3091	-
Kemiflex AB	Super Secu	PCP	2587	-
Kemiflex AB	Secu mellanbrun nr 2	PCP	2588	-
Nordsjö-Nordström & Sjögren AB	Inox Antimögel	PCP	2340	-
Tranemo Färg AB	Temonyl	PCP	2187	-
AB Texotan	Mystox LPL 100%	PCPL	3291	GB
AB Texotan	Mystox LSL	PCPL	3296	GB
AB Texotan	Mystox LSE	PCPL	3304	GB
AB Texotan	Mystox LE 50	PCPL	3311	GB
Färg AB International	Intertoxol T	PCPL	2737	-
Färg AB International	Intertox T	PCPL	2738	-

Data on companies, products, CPs and licenses from Produktkontrollnämnden, Protokoll 1977:6. ^†^ Generally, the CPs were mixtures, indicated CP-congener may not have been the major one in the product. TriCP and TetraCP usually correspond to 2,4,6-triCP and 2,3,4,6-tetraCP, respectively. PCPL stands for Pentachlorophenyllaurate. ^‡^ Origin (country) of product or label to the best of the authors´ knowledge, “-” means unknown (most likely SE). ^§^ BT stands for Bönnelyche & Thuröe, the company that originally produced Cuprinol products. Leading products for wood impregnation.

## Appendix B. Original Description of Odorous Houses, by SP

The text is a translated (by the authors) excerpt from an authority report from 1981 issued by The National Testing Institute (Statens Provningsanstalt, SP) which describes “Houses with mold odor” (Mögelluktande hus. Redovisning av skadefall. Teknisk Rapport 1981:37, ISSN 0280-2503). The underlined text was not included but appeared in an update from 1985 “Mold in houses” (Mögel i hus. Orsaker och åtgärder. Teknisk Rapport 1985:16, ISBN 91-7848-006-X).

“During the 1970s a completely new type of building damage started to occur that had previously not been heard of. Houses smelled like mold. Mold smell sneaked its way into the homes and gradually became increasingly apparent. Typically, the residents were not aware of the smell or didn’t want to recognize it, but friends and acquaintances told about it. Then came some high-profile cases where day nurseries and kindergartens were hit by mold odor. The children smelled when they came home and parents sounded the alarm. Several kindergartens were shut down and others rebuilt at high costs. During the late ‘70s and early ‘80s, more and more mold-smelling houses have been reported. If this is because damages are increasing or that residents now, thanks to intensive information through the press and radio/TV, themselves become aware of their problems, we cannot determine. What we do know is that the number of damages reported to us have increased substantially in recent years”—“A typical sequence of events looks like the following. A property owner or tenant is affected by mold odor. He notices the smell at certain weather types, when it is wet or when weather changes. The smell is pronounced along the skirting boards, but also in cupboards and wardrobes. The clothes smell, sheets and textiles smell even after washing. The smell is evident throughout the house if the dwelling has been closed for some time. He suspects moisture damages and makes a hole in the floor. There he finds no visible moisture and no visible mold growth. Everything looks dry but it smells like mold. Often the smell is in the mineral wool in the floor or in the floor mat. In this situation, he becomes unsure on how to proceed to get rid of the smell and, above all, how to get rid of the cause of the smell. The smell is in the floor, but the floor seems dry. He summons an expert, the builder for example, who, as it turns out, has so far never experienced this type of damage. He notes that the house was built according to documents and if he at all suggests any action it will be, for example, that the ventilation should be improved indoors. He may also suggest chemical decontamination of the floor construction, but he never suggests any radical change in the design. After the measures have been taken the smell comes back and new experts are summoned. Sometimes the case is taken to trial, sometimes it becomes an insurance case. New investigations are made and new proposals for action are proposed. In many houses around the country this description is recognized. Too many homes have been remediated at high costs but without results. This illustrates the scale of the problem. When trying to fix a mold smelling house one is in an area that is uncertain for many. One does not know the criteria for mold odor and can therefore make mistakes when taking measures”.
